# Population structure, sex ratio and growth of the seabob shrimp *Xiphopenaeus kroyeri* (Decapoda, Penaeidae) from coastal waters of southern Brazil

**DOI:** 10.3897/zookeys.457.6682

**Published:** 2014-11-25

**Authors:** Raphael Cezar Grabowski, Sabrina Morilhas Simões, Antonio Leão Castilho

**Affiliations:** 1 São Paulo State University (UNESP), Biosciences Institute of Botucatu, Zoology Department. Rubião Junior District, Botucatu, São Paulo, Brazil; 2 São Paulo State University (UNESP), Faculty of Sciences, Department of Biological Sciences. 14-01 Engenheiro Luis Edmundo Carrijo Coube Avenue, Vargem Limpa, Bauru, São Paulo, Brazil

**Keywords:** Asymptotic length, von Bertalanffy, longevity, Dendrobranchiata

## Abstract

This study evaluated the growth and population structure of *Xiphopenaeus kroyeri* in Babitonga Bay, southern Brazil. Monthly trawls were conducted from July 2010 through June 2011, using a shrimp boat outfitted with double-rig nets, at depths from 5 to 17 m. Differences from the expected 0.5 sex ratio were determined by applying a Binomial test. A von Bertalanffy growth model was used to estimate the individual growth, and longevity was calculated using its inverted formula. A total of 4,007 individuals were measured, including 1,106 juveniles (sexually immature) and 2,901 adults. Females predominated in the larger size classes. Males and females showed asymptotic lengths of 27.7 mm and 31.4 mm, growth constants of 0.0086 and 0.0070 per day, and longevities of 538 and 661 days, respectively. The predominance of females in larger size classes is the general rule in species of Penaeidae. The paradigm of latitudinal-effect does not appear to apply to seabob shrimp on the southern Brazilian coast, perhaps because of the small proportion of larger individuals, the occurrence of cryptic species, or the intense fishing pressure in this region. The longevity values are within the general range for species of Penaeidae. The higher estimates for longevity in populations at lower latitudes may have occurred because of the growth constants observed at these locations, resulting in overestimation of this parameter.

## Introduction

The impact of shrimp fisheries in tropical regions is now comparable to impacts on the world’s most intensively exploited temperate continental-shelf ecosystems. These fisheries have caused significant losses of spawning biomass and biodiversity, especially as a consequence of trawling on soft bottoms (Pauly and Christensen 1995, Pauly et al. 2002). Information about population biology can be important for understanding the life cycle of intensively fished species such as the seabob shrimp *Xiphopenaeus kroyeri* (Heller). This information can be developed from measurements of the size-class distribution, sex ratio, modal progression, growth, and longevity at spatial and temporal scales (Gab-Alla et al. 1990, Nakagaki and Pinheiro 1999).

*Xiphopenaeus kroyeri* has a wide geographical range in the western Atlantic Ocean, from Cape Hatteras (North Carolina, USA) to southern Brazil (Rio Grande do Sul) (Costa et al. 2007). Of the several species targeted by artisanal fishermen in southern Brazil, the seabob shrimp is one of the most important, and is also among the top ten penaeid species caught worldwide (Gillett 2008, Silva et al. 2013). Unfortunately, this species is overexploited in southeastern and southern Brazil (Vasconcellos et al. 2007, Almeida et al. 2012).

Many investigators have suggested that several environmental parameters and resources affect the observed patterns of population dynamics of species of decapod crustaceans. These parameters include temperature oscillations (proximate factor) and plankton productivity (ultimate factor), among others, and all are affected to various degrees by latitude (Bauer 1992, Clarke 1993, Boschi 1997, Castilho et al. 2007b, Costa et al. 2010).

Knowledge of the growth and longevity of penaeid shrimps is still limited, although these are important attributes in the study of population dynamics of heavily exploited vulnerable species (Petriella and Boschi 1997). The lack of studies on growth in decapod crustaceans can be attributed to difficulties in estimating growth, which are related to the absence of structures that can provide information about aging (Petriella and Boschi 1997, Branco 2005, Vogt 2012). Because growth in decapod crustaceans is discontinuous, frequently interrupted by successive ecdyses, the von Bertalanffy (1938) model is the most useful for the study of animals that grow rapidly, such as penaeid and sergestid shrimps (Petriella and Boschi 1997).

The present study evaluated the population biology of *Xiphopenaeus kroyeri* in the Babitonga Bay region, focusing on the sex ratio at different times of the year, juvenile recruitment, growth rates, and longevity of males and females. The longevity of *Xiphopenaeus kroyeri* was compared with studies of the same species at different latitudes, to determine whether the latitudinal paradigm is applicable to this population.

## Methods

### Study area

The sampling area of the present study is located in a subtropical region known as the Atlantic upwelling zone (from 23°S to 29°S). In the Atlantic Ocean, open ocean circulation is dominated by the opposing flow of the Brazil (subtropical) and the Malvinas (subantarctic) currents, which meet on average at 36°S (Acha et al. 2004). In southern Brazil, Babitonga Bay has an estuarine area surrounded by mangrove forests, and an adjacent marine environment with a high conservation priority (MMA 2007), and is a leading candidate for the creation of a sustainable-use marine protected area (MPA) (Vilar et al. 2011). The bottom sediment in the bay is composed mainly of sand, silty sand, and sandy silt, with little salinity stratification (IBAMA 1998). Tides are mixed and predominantly semi-diurnal (IBAMA 1998). During winter, the water temperature is vertically homogeneous, and during summer the cold water mass termed the South Atlantic Central Water (SACW) influences the region (Marafon-Almeida et al. 2008). The local fishing villages harvest crabs, shrimps, oysters, clams and fish, using gillnets, bottom trawling, long-lines, cast nets and the gerival (a tide/motor-driven net that targets shrimp) (IBAMA 1998, Pinheiro and Cremer 2003). The local fauna is poorly studied, particularly the seabob shrimp *Xiphopenaeus kroyeri*, despite its socioeconomic importance as attested by many investigators (Rodrigues 2000, Pinheiro and Cremer 2003, Branco 2005, Bail and Branco 2007).

### Biological sampling

**Figure 1.**
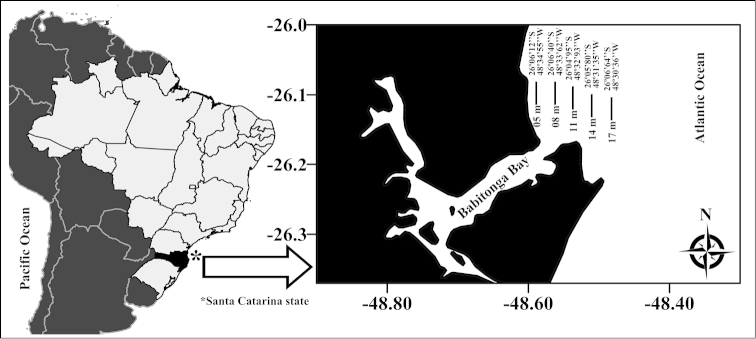
Map of the study area, Babitonga Bay, southern Brazil (Santa Catarina state), indicating locations and depths of the sampling sites.

Monthly trawls were conducted in the ocean adjacent to Babitonga Bay, off the municipalities of São Francisco do Sul and Itapoá on the northern coast of the state of Santa Catarina, using an artisanal shrimp fishing boat outfitted with double-rig nets (mesh size: 3 cm; total mesh gap: 11.5 m; boat velocity during trawls: 1.6 knots; total distance traveled during trawls: approximately 0.5 miles). Trawls were performed at five different depths (5, 8, 11, 14 and 17 m), sampling for 30 min at each depth, monthly, from July 2010 through June 2011 (five trawls per day, totaling 60 trawls in the year) (Fig. 1). For each point, latitude and longitude were taken with a GPS (Garmin GPSmap 76CSx), and depth was measured with an echobathymeter.

The carapace length (CL, to the nearest 1.0 mm), used as the standard measurement, includes the distance between the posterior margin of the eye orbit and the posterior margin of the carapace, and is widely used in studies of penaeid shrimps (Castilho et al. 2007a,b, 2008a, 2012, Heckler et al. 2013). Size-frequency distributions were constructed using size classes of 1 mm CL.

### Sex ratio

The sex of individuals was determined by the presence (males) or absence (females) of petasma. The sex ratio was estimated as the quotient between the number of males and the total number of individuals in samples from each month. Deviations from a 1:1 sex ratio were tested using a binomial test (α = 0.05) (Wilson and Hardy 2002, Baeza et al. 2013). After the sexes were sorted, individuals were classified as juvenile (sexually immature) or adult. Juvenile females were considered as those with ovaries ranging from thin to thicker, transparent strands; adult females were categorized by the color and volume occupied by the gonads (Castilho et al. 2008a). Adult males were identified by their fused petasma, and juvenile males by separated petasma (Boschi 1989).

### Individual growth and longevity

Growth and longevity were analyzed for males and females separately (Boschi 1969), based on the von Bertalanffy growth model (von Bertalanffy 1938) and using the methodology adopted by Simões et al. (2013). Modal values were determined for each CL frequency using the software PEAKFIT (Automatic Peak Fitting Detection and Fitting, Method I-Residual, no Data Smoothing), with size classes of 1.0 mm, according to Fonseca and D’Incao (2003). The models were plotted on a scatter graph vs. age, to analyze the growth rhythm of the cohorts. Growth parameters (*CL_∞_*: asymptotic carapace length; *k*: growth coefficient (day^-1^); *t_0_*: theoretical age at size zero) were estimated by using the SOLVER supplement in Microsoft Excel (version 2010) for Windows 7, which applies the Von Bertalanffy growth model: *CL_t_*=*CL_∞_*[1-exp*^-k^*^(^*^t-t0^*^)^] (*CL_t_*: carapace length at age *t*). The growth of a cohort was evaluated based on its similarity to values previously observed for this species (Campos et al. 2011, Heckler et al. 2013). Cohort data were pooled and growth parameters were estimated. The estimated growth curves for males and females were compared by F test (*p*=0.05) (Cerrato 1990). Longevity was calculated using the inverse von Bertalanffy growth model, with a modification suggested by D’Incao and Fonseca (1999), which is given by: longevity= 0-(1/*k*)Ln[1-(*CL_t_*/*CL_∞_*)] (considering *t_0_*= 0, and *CL_t_*/*CL_∞_*= 0.99).

## Results

### Population structure

Throughout the sampling period, 4,007 specimens of *Xiphopenaeus kroyeri* were examined, including 1,722 males and 2,285 females (43% and 57%, respectively) of which 2,901 were adults and 1,106 juveniles. Carapace length ranged from 7.1 to 29.7 mm for males and 6.0 to 31.8 mm for females.

**Figure 2.**
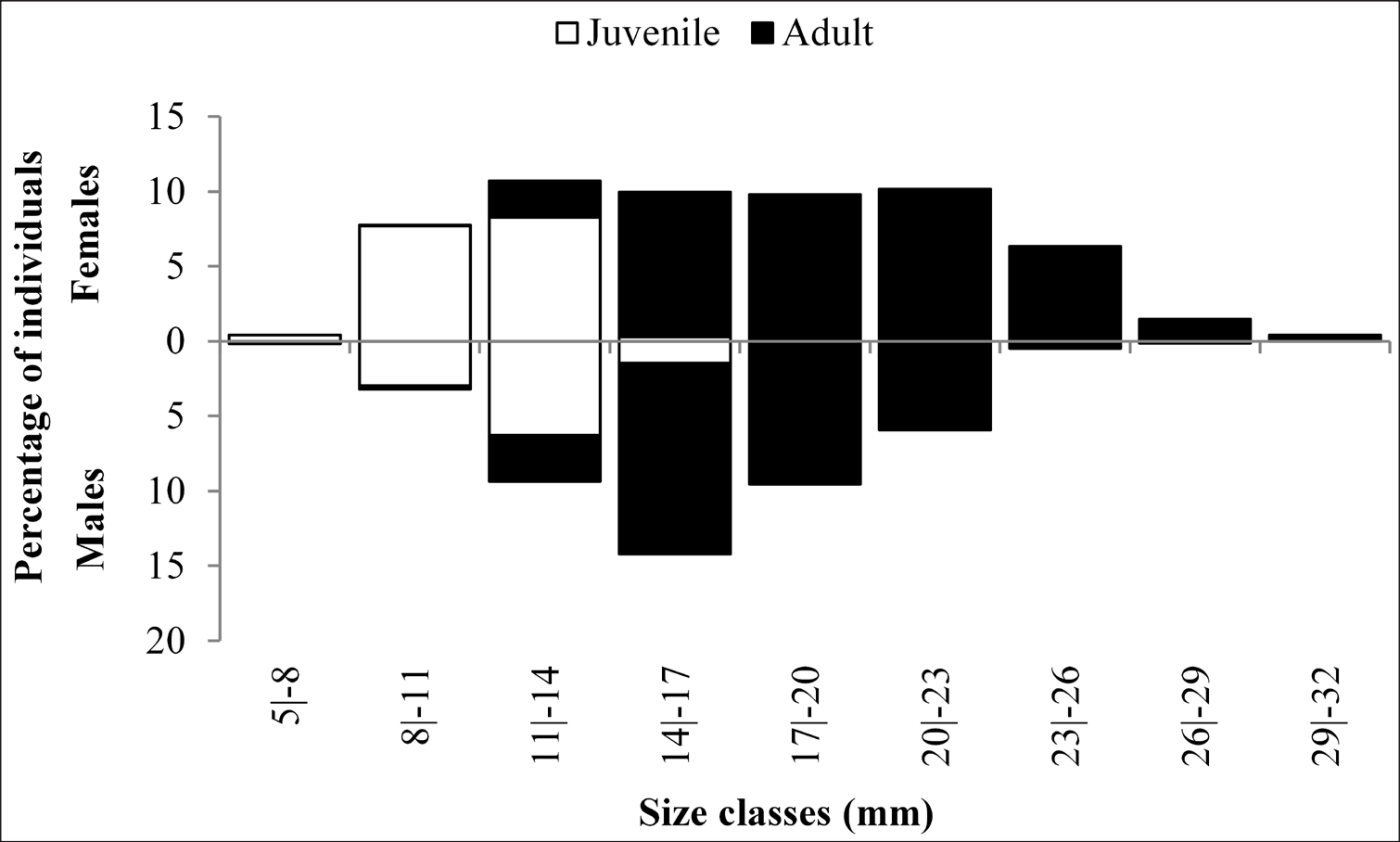
*Xiphopenaeus kroyeri*. Distribution of the percentage of juveniles and adults by size classes (CL, mm) observed for individuals collected from July 2010 through June 2011 in an area adjacent to Babitonga Bay, southern Brazil.

Among adults, males were more abundant in the size classes between 8.0 and 16.9 mm. Beginning in the 17.0–19.9 mm size class, adult females were more abundant, with a peak in the 20.0–22.9 mm size class. Juvenile individuals occupied the lower size classes, predominating in the 11.0 to 13.9 mm size class; and were not found in size classes larger than 17.0–20.0 mm (Fig. 2).

### Sex ratio

**Figure 3.**
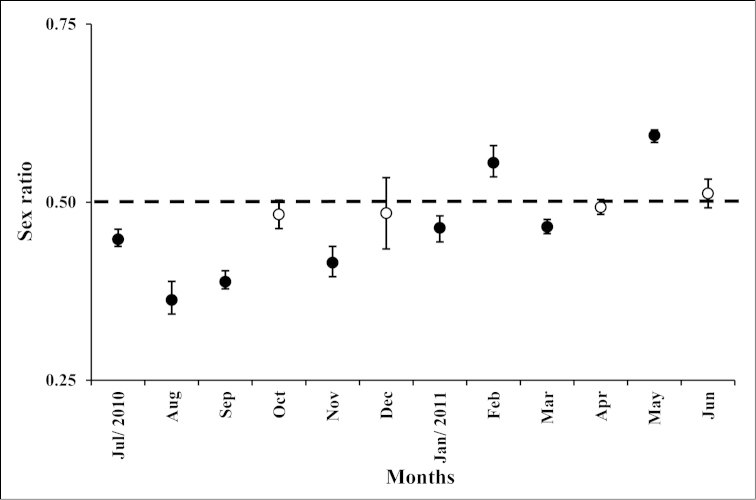
*Xiphopenaeus kroyeri*. Monthly sex ratio (estimate ± standard error) of adults collected from July 2010 through June 2011 in an area adjacent to Babitonga Bay, southern Brazil. Black circles indicate significant deviation from a 1:1 sex ratio (Binomial test, *p* < 0.05).

During the study period, we observed a mean sex ratio of 0.5. Female-biased sex ratios (<0.5) were observed in July, August, September, November, January and March, while male-biased sex ratios (>0.5) were obtained in February and May (Fig. 3).

### Individual growth and longevity

**Figure 4.**
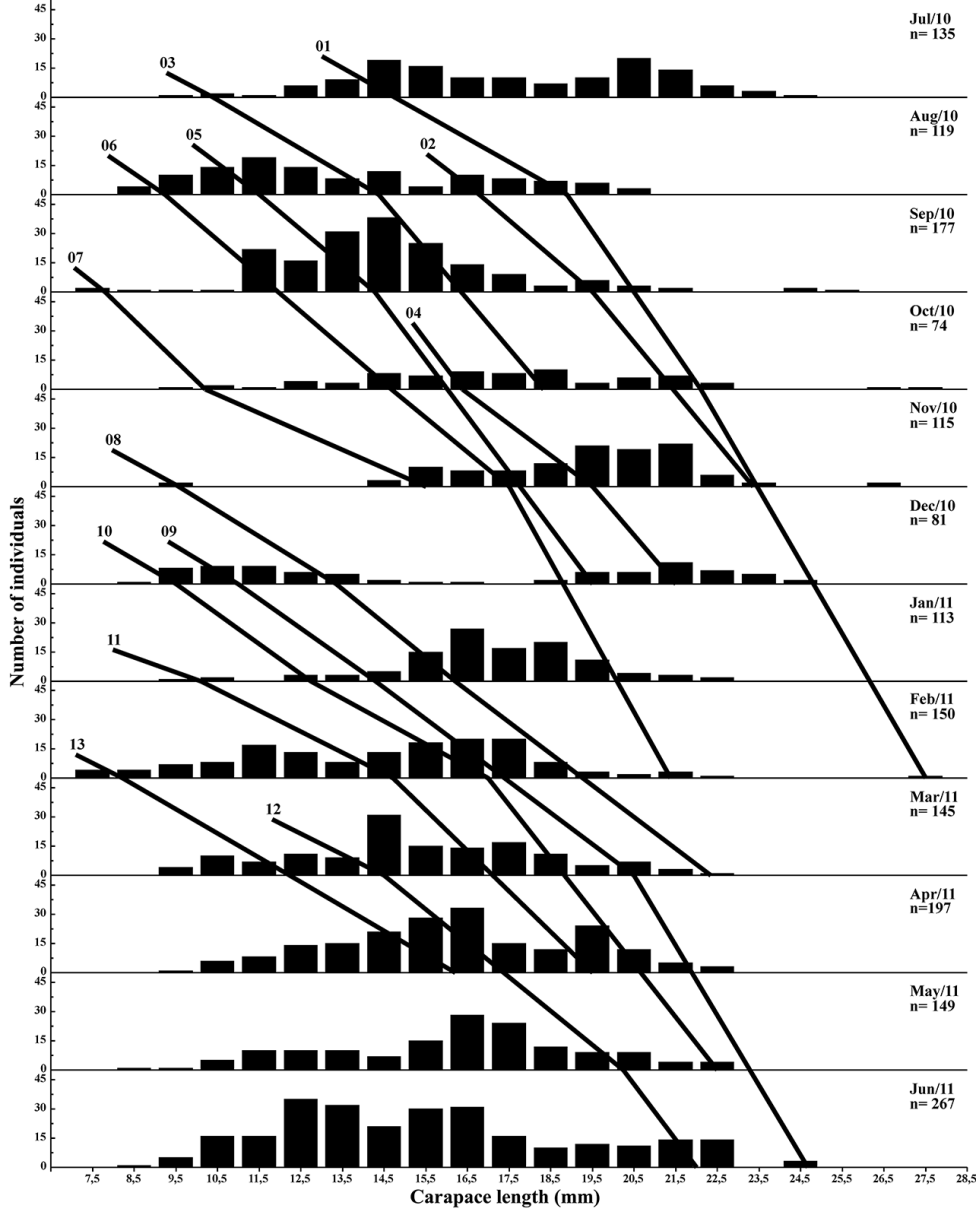
*Xiphopenaeus kroyeri*. Selected cohorts for growth analysis and number of males collected each month from July 2010 through June 2011 in an area adjacent to Babitonga Bay, southern Brazil.

**Figure 5.**
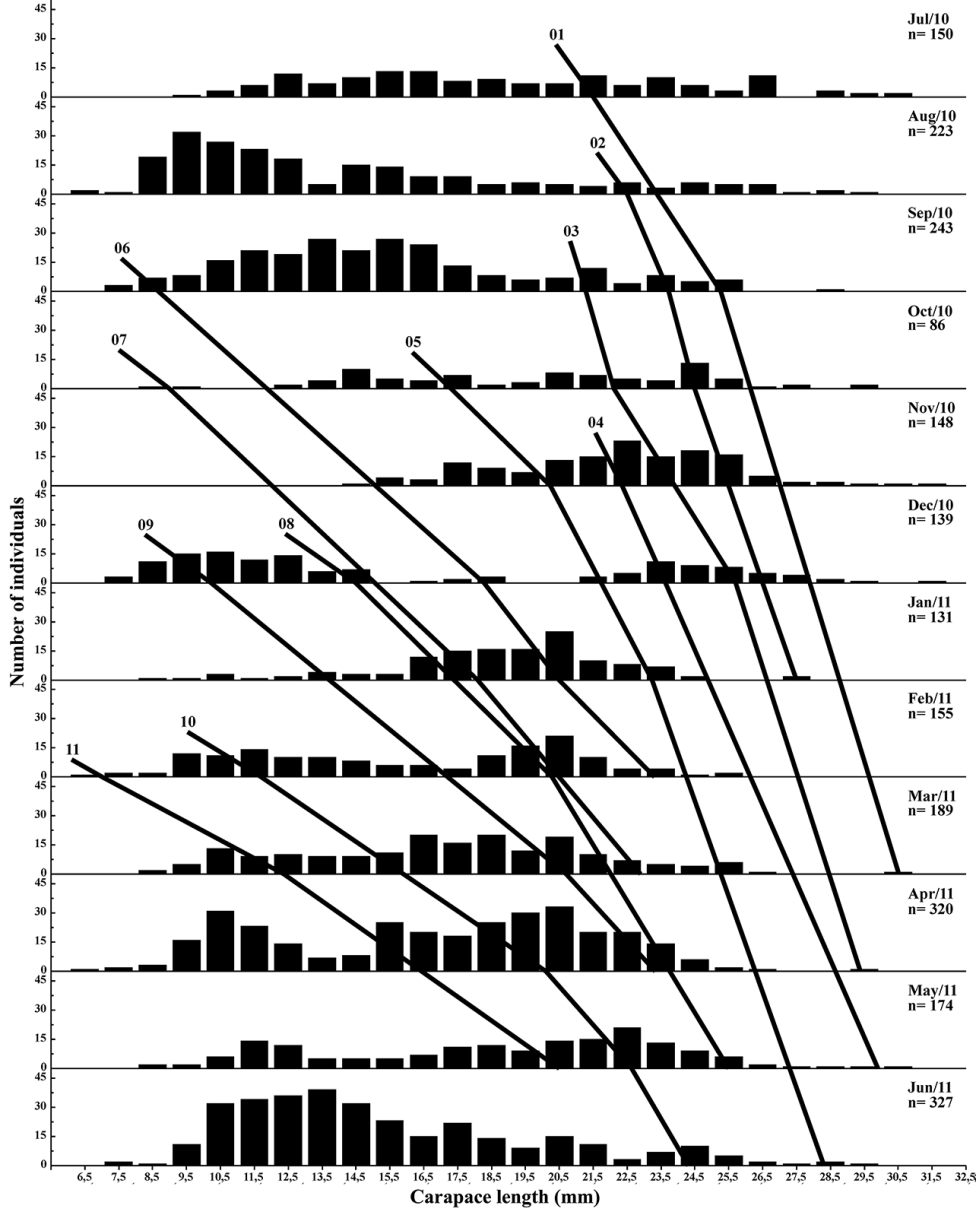
*Xiphopenaeus kroyeri*. Selected cohorts for growth analysis and number of females collected each month from July 2010 through June 2011 in an area adjacent to Babitonga Bay, southern Brazil.

**Figure 6.**
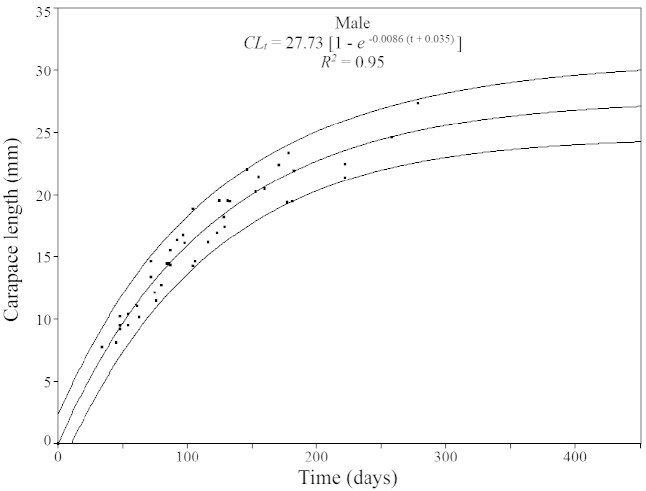
*Xiphopenaeus kroyeri*. Mean growth curve estimated for males collected in an area adjacent to Babitonga Bay, from July 2010 through June 2011, based on the von Bertalanffy growth model. Outer lines: 95% prediction interval.

**Figure 7.**
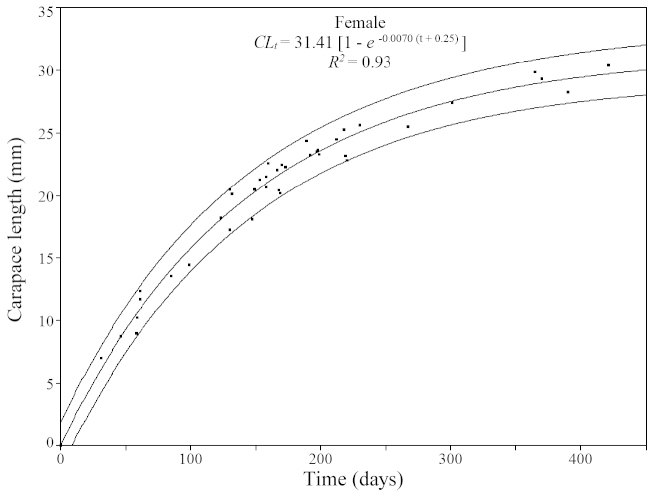
*Xiphopenaeus kroyeri*. Mean growth curve estimated for females collected in an area adjacent to Babitonga Bay, from July 2010 through June 2011, based on the von Bertalanffy growth model. Outer lines: 95% prediction interval.

Based on the modal values, 13 cohorts for males (Fig. 4) and 11 cohorts for females (Fig. 5) were selected. An overall mean growth curve was constructed, grouping the cohorts obtained for males and females separately (Figs 6, 7). Based on these curves, overall growth models were determined for each gender, which resulted in estimates of *CL_∞_*= 27.73 mm, *k* = 0.0086/day (3.14/year) and *t_0_*= -0.035 for males and *CL_∞_*= 31.41 mm, *k* = 0.0070/day (2.56/year) and *t_0_*= -0.25 for females.

Longevity was estimated to be 538 days (or 1.47 years) for males and 661 days (or 1.81 years) for females. Growth curves for males and females showed a significant difference (*F_calculated_*= 3.157 > *F_critical_*= 2.695; *p* = 0.028; degrees of freedom: 3/101).

## Discussion

For Dendrobranchiata, results of several studies have revealed a predominance of females in higher size classes, e.g., for *Artemesia longinaris* Bate (Castilho et al. 2007a, Costa et al. 2010), *Pleoticus muelleri* (Bate) (Castilho et al. 2008a, Dumont and D’Incao 2008) and *Sicyonia dorsalis* Kingsley (Castilho et al. 2008b), as well as for *Xiphopenaeus kroyeri* (Branco 2005, Castro et al. 2005, Almeida et al. 2012, Eutrópio et al. 2013, Heckler et al. 2013). These studies also collected overall more females than males, which concords with results of the present study. In penaeid shrimps, females generally reach larger sizes than males (Boschi 1969, 1989). As observed by Gab-Alla et al. (1990), Branco et al. (1999) and Yamada et al. (2007), this difference may be associated with the reproductive biology, as an increase in carapace length indicates a capacity to produce more oocytes, with a consequent increase in fecundity.

Several studies regarding *Xiphopenaeus kroyeri* (Branco et al. 1994, Nakagaki and Negreiros-Fransozo 1998, Lopes et al. 2010) reported male-biased sex ratios. These fluctuations can be attributed to segregated distributions of the sexes at certain times of year (Signoret 1974, Branco 2005). Alternatively, a female-biased sex ratio may be associated with higher mortality in males (Cha et al. 2002). Kevrekidis and Thessalou-Legaki (2006), studying *Melicertus kerathurus* (Forskål), stated that the high metabolic demand of females during the period of gonadal maturation forces them to feed for long periods, increasing their vulnerability to sampling gear. Lopes et al. (2010) stated that a male-biased sex ratio is less common in *Xiphopenaeus kroyeri*, and might be associated with migration of females to greater depths during spawning periods.

Gender is among the factors that influence growth in Penaeoidea (Petriella and Boschi 1997). Generally, males of Penaeidae show higher growth coefficients than females, resulting in lower asymptotic lengths, and males are smaller than females of equal age. In this study, females showed a lower growth coefficient and greater asymptotic length, which concords with information provided by García and Le Reste (1986) and Petriella and Boschi (1997), as well as with previous analyses of the growth of this species along the Brazilian coast (Branco 2005, Fernandes et al. 2011, Heckler et al. 2013).

On the Brazilian coast, the growth of *Xiphopenaeus kroyeri* has been studied by Santos and Ivo (2000) on the coast of the state of Bahia; Heckler et al. (2013) off São Paulo; and Campos et al. (2011) off Santa Catarina, among others. Santos and Ivo (2000) obtained the lowest *k* values, as well as the highest asymptotic length (females: *k* = 0.767/year, *CL_∞_*= 37.2 mm; males: *k* = 0.986/year, *CL_∞_*= 28.0 mm), followed by Campos et al. (2011) (females: *k* = 2.628/year, *CL_∞_*= 31.0 mm; males: *k* = 2.993/year, *CL_∞_*= 28.0 mm) and by Heckler et al. (2013) (females: *k* = 2.190/year, *CL_∞_*= 30.54 mm; males: *k* = 3.280/year, *CL_∞_*= 26.38 mm). Castilho et al. (2007b) suggested that several aspects of the life cycle such as longevity, body size, and size at the onset of sexual maturity show variations related to environmental factors that can be associated with latitude. As latitude increases, individuals tend to grow larger and more slowly, probably because of the colder water temperatures. This is known as the latitudinal effect paradigm, which has been analyzed with respect to its influence on population dynamics of penaeid shrimps by several researchers (Bauer 1992, Boschi 1997, Costa and Fransozo 2004, Castilho et al. 2007b, Costa et al. 2010).

The results of growth analyses for *Xiphopenaeus kroyeri* along the southern Brazilian coast do not seem to follow the latitudinal effect pattern. The lower growth coefficient (and consequently the greater asymptotic length) observed by Santos and Ivo (2000) can be explained by the methodology adopted by the authors, who used the software FAO-ICLARM Stock Assessment Tool (FISAT), which includes the ELEFAN routine (Electronic Length Frequency Analysis). Fonseca (1998) noted that this software tends to exclude extreme lengths from the analysis, which causes errors in estimating the growth coefficient. The analysis conducted by Freire (2005) on the São Paulo coast (Ubatuba) provided higher results for asymptotic length, compared to that obtained by Campos et al. (2011) on the Santa Catarina coast and to the present results. These differences can be explained by differences in sampling depth, since the study by Freire (2005) included deeper sites (up to 35 m). The results obtained in the present growth analyses are consistent with the maximum size of the shrimp observed in field sampling (females: estimate = 31.41 mm, field observation = 31.80 mm; males: estimate = 27.73 mm; field observation = 27.90 mm).

Three additional hypotheses may explain the disagreement here presented in relation to the latitudinal effect pattern. First, as observed by Gusmão et al. (2006), it is possible that two different species of *Xiphopenaeus* live in the southwestern Atlantic. In the brazilian coast, these authors examined an area extending from Natal in the state of Rio Grande do Norte in northeastern Brazil, to Ubatuba, and found that *Xiphopenaeus* sp. 1 occurred throughout the area, whereas *Xiphopenaeus* sp. 2 showed a discontinuous distribution, with recorded occurrences only at Natal and Ubatuba. The second hypothesis is based on the number of individuals sampled in higher size classes, which can be explained in many ways. As noted by Campos (2006), larger animals could be less vulnerable to capture, and may have some ability to escape from the sampling gear. The author also suggested that larger individuals migrate to areas that are not accessible to the artisanal fishery. In agreement with this suggestion, Freire (2005) observed an increasing trend in CL toward greater depths, explaining the higher CL values found in his study. In further support of the difference in efficiency of the fishing methods, in 2011, even with a smaller fleet (14% of 806 vessels), the industrial fishery accounted for 47% of all seabob shrimp landings in the state of São Paulo (Mendonça et al. 2013).

The third hypothesis considers the intense fishery effort off the coast of Santa Catarina, which has been studied by D’Incao et al. (2002), Branco (2005) and Vasconcellos et al. (2007), among others. Although Mendonça et al. (2013) reported that the seabob shrimp stocks off São Paulo state have stabilized in recent years, Vasconcellos et al. (2007) argued that these stocks off southeastern and southern Brazil are overexploited. This intense fishery exploitation was responsible for the decrease in yield of the seabob shrimp fishery in these areas (D’Incao et al. 2002). It is entirely possible that, due to the intense fishery effort in this area, the stocks cannot maintain their normal population structure, i.e., individuals cannot reach larger size classes.

The absence of a pattern of asymptotic length estimated for this species may be a consequence of gene flow between the populations off Santa Catarina and São Paulo, since under favorable conditions the shrimp can migrate up to 900 km, as observed by Fenucci (1988) for *Melicertus plebejus* (Hess) on the Australian coast. Also, similarities in reproductive biology among populations that are in some cases located thousands of kilometers apart, may indicate the existence of an open meta-population, with considerable connectivity in the southwestern Atlantic (Almeida et al. 2012).

The present results for the longevity and growth coefficient of *Xiphopenaeus kroyeri* fall within the range proposed by García and Le Reste (1986) and Pauly (1984) for these parameters in penaeid shrimps. These investigators found that the shrimp complete their life cycle in approximately two years, with growth coefficients between 0.25 and 2.5. Our estimates suggest that, the seabob shrimp in the study area has a longevity of 538 days or 1.47 years for males, and 661 days or 1.81 years for females. Similarly, Campos et al. (2011) on the coast of Tijucas, Santa Catarina, estimated a longevity of 561 days for males and 641 days for females. Analyses of populations at lower latitudes have estimated greater longevities for males and females respectively: 1705 and 2192 days (Santos and Ivo 2000), 2192 and 2422 days (Santos 1997) and 1535 and 1212 days (Santos 1997). According to D’Incao and Fonseca (1999), most longevity analyses for penaeid shrimps have resulted in overestimates; based on underestimates for the growth coefficient, which increases the longevity estimate.

## Conclusion

In general, the present results indicate that *Xiphopenaeus kroyeri* completes its life cycle within the study area, because juveniles as well as adults with a range of sizes were collected. All estimates in our study concord with current knowledge of the life cycle of *Xiphopenaeus kroyeri*, are within the ranges proposed by several investigators, and are similar to values observed in field sampling. This study provides a theoretical basis for informed management of this fishery along the Brazilian coast.
